# Upregulation of Circulating *MiR-21* Expression as a Potential Biomarker for Therapeutic Monitoring and Clinical Outcome in Breast Cancer

**DOI:** 10.31557/APJCP.2019.20.4.1223

**Published:** 2019

**Authors:** Sumadi Lukman Anwar, Dwi Nur Indah Sari, Aprilia Indra Kartika, Meutia Srikandi Fitria, Dewi Sahfitri Tanjung, Dinna Rakhmina, Tirta Wardana, Indwiani Astuti, Sofia Mubarika Haryana, Teguh Aryandono

**Affiliations:** 1 *Division of Surgical Oncology, Department of Surgery, *; 2 *Graduate Program, *; 7 *Departement of Pharmacology and Therapy,*; 8 *Department of Histology and Cell Biology, Faculty of Medicine, Public Health and Nursing, Universitas Gadjah Mada, Yogyakarta,*; 3 *Faculty of Health Sciences, Setia Budi University, Surakarta, *; 4 *Medical Laboratory Technology, Health and Nursing Faculty, Universitas Muhammadiyah Semarang, Semarang, *; 5 *Current position: PT Etana Biotechnologies Indonesia, Jakarta, *; 6 *Politeknik Kesehatan Kemenkes Banjarmasin, Banjarmasin, Indonesia.*

**Keywords:** MicroRNA, miR-21, breast cancer, biomarker

## Abstract

**Background:**

Aberrant patterns of microRNA expression have been highlighted as a potential clinical biomarker in breast cancer as the most frequent cancer among women that contributes nearly a quarter of total cancer incidence in 2018. Upregulation of microRNA-21 (*miR-21*) is associated with adverse clinical outcomes in breast cancer. However, the use of circulating free *miR-21* as a non-invasive biomarker for diagnosis and therapeutic monitoring in breast cancer is not well established. We quantified the levels of circulating *miR-21* expression and analyzed their correlation with clinicopathological variables and progression-free survival.

**Materials and Methods:**

This initial study included a cohort of 102 breast cancer patients of different subtypes and clinicat stages. We also included 15 unrelated healthy women. Venous blood from patients was collected at diagnosis and after treatment of surgery and chemotherapy. *MiR-21* expression was quantified from total RNA fraction isolated from patient’s plasma. Quantitative reverse transcription polymerase chain reaction (qRT-PCR) was used to analyzed *miR-21* expression.

**Results:**

Expression of circulating *miR-21* was significantly elevated in breast cancer patients compared to healthy women (median *miR-21* expression levels were 7.67±2.2 and 1.28±0.16, respectively; p<0.0001). Significant reduction of *miR-21* expression was observed in breast cancer patients after completion of surgery and chemotherapy (median *miR-21* expression levels were 7.67±2.2 at diagnosis and 2.16±1.28 after treatment, respectively; p<0.0001). *MiR-21* expression was higher in breast cancer patients younger than 40-year-old but was not significantly different according to different histopathological grades and clinical stages at diagnosis. Patients with upregulation of circulating *miR-21* were associated with poor progression-free survival (median survival 72 vs 86 weeks, respectively; log-rank (Mantel-Cox) test, p=0.049).

**Conclusion:**

*MiR-21* expression was upregulated in breast cancer patients and might serve as a therapeutic monitoring marker.

## Introduction

Breast cancer has posed as a major public health challenge because the incidence is continuously increasing during the past decades (Bray et al., 2018). With more than 2 million new cases in 2018, breast cancer is the most frequent cancer diagnosed among women worldwide including in Indonesia (Bray et al., 2018). Although significant improvement in the diagnosis and treatment has recently been achieved, breast cancer-related mortality and morbidity are relatively high particularly in low- and middle-income countries (Aryandono et al., 2006; Youlden et al., 2012). In Indonesia, breast cancer patients often present in late stages due to lack of public awareness, screening participation, as well as access for diagnosis and treatment (Anwar et al., 2018a; Anwar et al., 2018b). In addition, breast cancer is a heterogeneous disease showing various phenotypes, subgroups, and different responses to treatment (Perou and Borresen-Dale, 2011; Widodo et al., 2017). Cancer antigen 15-3 (CA-153) is currently the most common biomarker used in breast cancer although the relevance into the clinical practice, outcome, and monitoring of relapse are still controversial due to lacking of sensitivity and specificity (Atoum et al., 2012). Therefore, investigation for finding novel non-invasive biomarker in breast cancer is required.

MicroRNAs are small non-coding RNAs regulating gene expression post-transcriptionally through direct interaction with the messenger-RNA targets (Croce, 2009). Deregulation of particular microRNA panels has been associated with diagnostic and prognostic values of various cancers including breast cancer (Croce, 2009; Volinia et al., 2012). Patterns of microRNA expression have also been extrapolated in breast cancer sub-classification (Bockmeyer et al., 2011) and metastasis detection (Wang and Wang, 2012). In the rapidly expanding field of cancer stem cells, several microRNAs have also been reported in maintaining the stemness phenotypes (Shimono et al., 2009). We recently reported interaction of a polymorphism with microRNA binding and downregulation of target gene expression as well as their correlation with tumor size and lymph node infiltration in estrogen receptor (ER)-negative breast cancer (Anwar et al., 2017). In addition, microRNAs are also released from tumor cells into blood circulation through lipid vesicles, microvesicles, exosomes, and apoptotic bodies (Turchinovich et al., 2012). Circulating microRNAs typically appear in a complex formation with ribonucleoprotein or high-density lipoprotein owing their stability against endogenous RNase degradation (Roth et al., 2010). Detection of circulating microRNAs has also gained interest in its potential application as a non-invasive biomarker in cancer (Roth et al., 2010). 

*MiR-21* is one of the most frequently reported oncogenic microRNA in various solid tumors in which the upregulation is commonly related with adverse outcome (Dillhoff et al., 2008; Lee et al., 2011; Zaman et al., 2012). Functional studies have also shown that *miR-21* upregulation is able to induce cell proliferation, migration, and inhibit apoptosis (Si et al., 2007; Yang et al., 2011). Validated *miR-21* target genes are mostly tumor suppressor genes including* PTEN* (Dai et al., 2017) and *PDCD4 *(Frankel et al., 2008). Although prognostic and diagnostic values of *miR-21* in breast cancer have been highlighted in some reports (Lee et al., 2011; Pan et al., 2014), the debate regarding the cost-effective measurement and practical implementation remains exist. In addition, exploration of *miR-21* alteration after therapy is underreported. In this study, we investigated the potential use of circulating *miR-21* as a biomarker for diagnosis, prognosis and therapeutic monitoring.

## Materials and Methods

*Patient cohort*

Blood samples were collected in the periode of 2014-2015 from 102 breast cancer patients at diagnosis, 40 breast cancer patients after completion of surgery and chemotherapy, and 15 healthy women without a history of benign and malignant tumors. Patients and participants who were older than 18 years old, were confirmed diagnosis by histopathology, and were able to provide inform concent, were then recruited. Vulnerable patients and participants such as with dementia and terminally-ill patients were excluded. Tumor stage and clinicopathology variables were collected at the time of diagnosis. Follow up of patients was performed according to the local clinical recommendation. Progression-free survival was defined as the period from the time at diagnosis to any relapse or local/regional/distant disease spreading observed until June 2017. Table 1 summarized the clinicopathology variables of patients included in this study. Stage I-II of breast cancer are often classified as early stage and stage III-IV are considered as advanced / late stage. 

Sample size was estimated using effect size of Cohen’s approach with α=0.05, moderate effect size of 0.5, and statistical power of 0.9. The healthy individuals used as controls were selected randomly to represent the population. Protocol of the study was approved by the Medical and Health Research Ethics Committee of the Faculty of Medicine, Universitas Gadjah Mada, Indonesia (601/EC/2014). All participants gave written informed concent prior to participating in this study.

*RNA isolation from plasma*

The peripheral venous blood samples were collected using EDTA-vacutainer collection tubes and centrifugated at 1500 rpm for 10 minutes. The supernatant plasma was stored at -80^o^C until analysis. Total RNA was isolated from 200 µL plasma using miRCURYTM RNA Isolation Kit-Biofluid (Exiqon, Cat No. 300112) according to the manufacturer’s recommendation. The plasma was mixed with Lysis and Protein Precipitation Solution. After centrifugation 12,000 rpm, the supernatant was mixed with 270 µL isopropanol and was then applied into microRNA Mini Spin Column (Exiqon). After washing steps, total RNA was eluted using 25 µL RNA-se free water. For internal control during RNA extraction, we used spike-in of Sp6 into each plasma sample. 

**Table 1:** Clinicopthological Variables of Breast Cancer Patients (N=102) and the Correlation with Circulating miR-21 Expression

Variables/ Category	N	miR-21 expression (mean±SE)	p-value
Age			
> 40 year-old	84	29.7±5.6	0.03
≤ 40 year-old	18	21.5±4.66	
Tumor differentiation			
Grade I	9	23.5±7.0	0.09
Grade II	44		
Grade III	49	21.8±2.87	
Tumor size			
> 5 cm	45	24.5±5.1	0.77
≤ 5 cm	57	21.3±4.7	
Stage at diagnosis			
Stage I	3	20.91±3.33	0.54
Stage II	45		
Stage III	51	24.23±6.76	
Stage IV	3		
Subtype			
Luminal	9	22.6±3.2	0.98
Her2	56		
Triple-negative	27	22.8±3.5	

**Figure 1:**
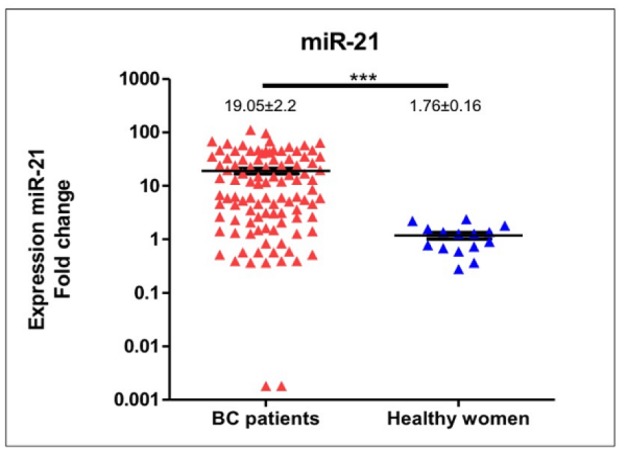
Frequent Upregulation of Circulating miR-21 Expression Levels in Breast Cancer Patients Compared to Healthy Women

**Figure 2:**
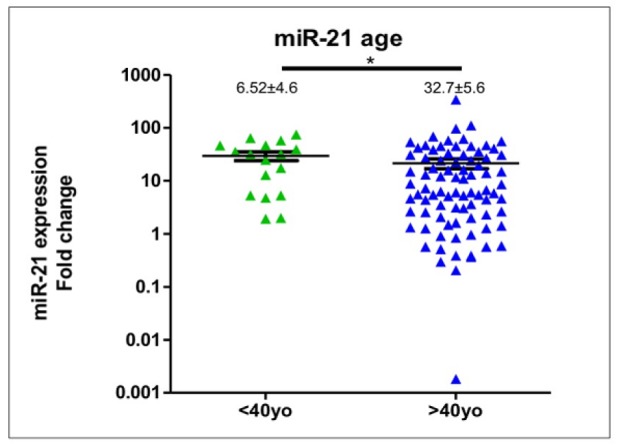
Expression of Circulating miR-21 in Breast Cancer Patients Younger and Older than 40 Year-Old. Breast cancer patients at or younger than 40 year-old showed higher circulating miR-21 expression compared to the older counterparts

**Figure 3:**
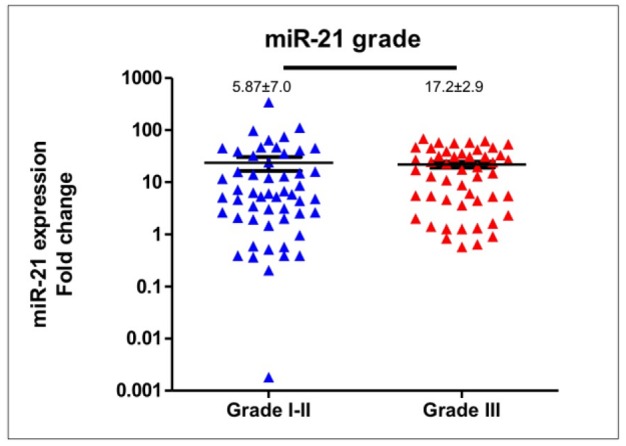
Correlation of Expression of Circulating miR-21 and Tumor Differentiation. miR-21 expression levels were not significantly different according to the tumor grade

**Figure 4:**
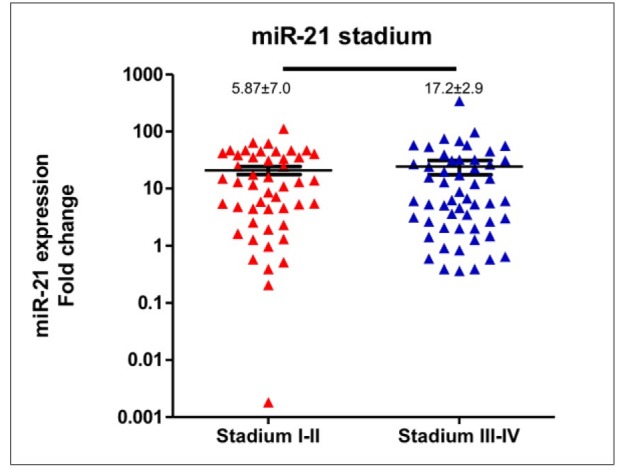
Correlation of Expression of Circulating miR-21 and Stages at Diagnosis. miR-21 expression levels were not significantly different in early and late stage breast cancer patients

**Figure 5:**
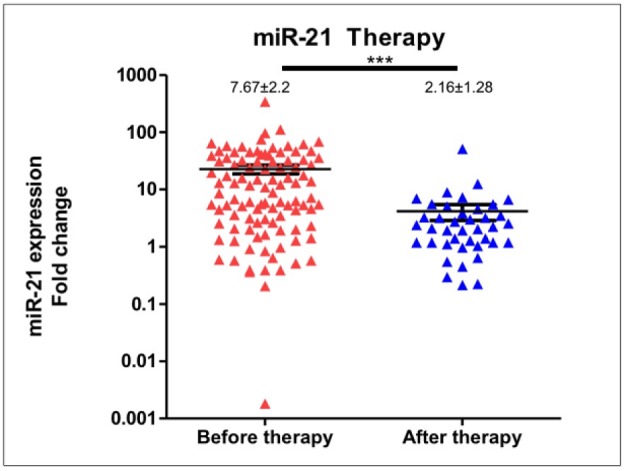
Decreased Expression of Circulating miR-21 in Breast Cancer after Surgery and Chemotherapy. Compared to the baseline, miR-21 expression was significantly lower after completion of therapy

**Figure 6:**
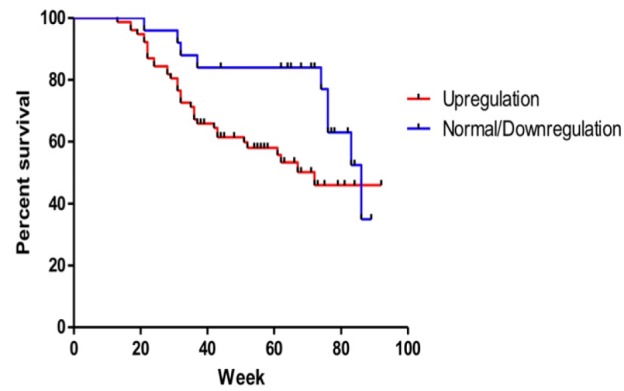
Upregulation of Circulating miR-21 Correlated with Lower Progression-Free Survival. MiR-21 upregulation in breast cancer patients is associated with shorter progression-free survival (median PFSs were 72 vs 86 weeks, Mantel-Cox test p=0.049)

*Quantification of miR-21 expression*

Enriched RNA from plasma was reverse transcribed using Universal cDNA Synthesis kit II, 8-64 rxns (Exiqon, Cat No.203301) in a total reaction volume of 20 µL. *MiR-21* expression levels were measured using ExiLent SYBR Green master mix (Exiqon, Cat No. 203402) and specific primers for *miR-21* (Exiqon, Cat YP00204230), Sp6 (Exiqon, Cat YP00203954), and MIR-16 (Exiqon, YP00205702). 

*Statistical analysis*

The relative expression of *miR-21* was normalized using spike-in Sp6 and miR-16 to further be compared with the expression in healthy individuals. Fold change expression was calculated using the 2^-ΔΔCq^ method. The expression levels of *miR-21* between breast cancer patients and healthy women were compared using the Mann-Whitney-U test. The *miR-21* expression levels at diagnosis and after treatment were compared and the correlation between clinicopathologic variables with *miR-21* expression was also be compared with Mann-Whitney-U tests. Upregulation was determined as mean of *miR-21* expression + 2xSD. Kaplan-Meier plot and Log-rank (Mantel-Cox) test were used for survival analysis. All statistical tests were 2-sided and a p-value < 0.05 was designated as statistically significant. Statistical analysis was performed using GraphPad Prism (La Jolla CA, USA).

## Results

*Elevated expression levels of circulating miR-21 in breast cancer patients*

In compared to healthy women (n=15), expression levels of circulating *miR-21* in breast cancer patients (n=102) was significantly higher (median expression levels were 7.67±2.2 and 1.28±0.16, respectively; p<0.0001), please see Figure 1. *MiR-21* expression levels in patients were deregulated with inter-tumoral great variations reflecting tumor heterogeneity and somatic tumor evolution. We used spike-in both Sp6 and MIR-16 to normalize the *miR-21* expression. Comparing *miR-21* expression in patients younger and older than 40 years-old revealed a significant difference (median expression levels were 32.07±5.6 and 6.52±4.58, respectively; p=0.02), Figure 2. *MiR-21* expression in breast cancer patients with histopathological good/moderate (Grade I-II) and poor (Grade III) differentiation was not significantly different (median expression levels were 5.87±7.04 and 17.2±2.9, respectively; p=0.09), Figure 3. In addition, *miR-21* expression in early stage (Stadium I-II) and late stage (Stadium III-IV) was not significantly different (median expression levels were 12.38±3.3 and 6.18±6.76, respectively; p=0.54), Figure 4. *MiR-21* expression levels were also not significantly different according to tumor sizes and breast cancer subtypes (Table 1).

*Expression of miR-21 after surgery and chemotherapy*

Quantification of *miR-21* expression in breast cancer patients that have completed surgery and chemotherapy was performed in 40 patients. Compared to the expression at diagnosis, *miR-21* expression was significantly lower after completion of therapy (median expression levels were 7.67±2.2 and 2.16±1.28, respectively; p<0.0001), Figure 5.

*Correlation of circulating miR-21 upregulation with progression-free survival*

Upregulation of *miR-21* expression at diagnosis was determined as an average *miR-21* expression in healthy women plus 2xSD as described at the Material and Methods. Progression-free survival was compared between patients with and without *miR-21* upregulation at diagnosis. Patients with *miR-21* upregulation showed shorter progression-free survival, median survivals were 72 vs 86 weeks and Mantel-Cox test p=0.049, Figure 6. 

## Discussion

We presented the potential roles of circulating *miR-21* as therapeutic monitoring and prognosis biomarkers in breast cancer. Circulating *miR-21* expression was significantly lower after surgery and chemotherapy and the upregulation was associated with shorter PFS (Figure 5 and 6). An emerging area in breast cancer study is the development of a new biomarker for prediction of therapeutic response and prognosis. MicroRNA deregulation has been associated with certain clinicopathological variables and outcome in breast cancer (Volinia et al., 2012). Gene expression in primary tumor specimens might reflect the biology of the tumor and has been referred as a prognostic biomarker with substantial performance (Croce, 2009; Iorio and Croce, 2012). As microRNAs are released from tumor cells using different mechanisms, detecting particular microRNAs in circulation or body fluid might resemble pathobiology and distinct characteristics of the tumor (Turchinovich et al., 2012). In addition, the relative stability against varying temperature and pH and the presence in body fluids including plasma, urine, and saliva have corroborated microRNA as a solid candidate for a minimally-invasive cancer biomarker (Schwarzenbach et al., 2014). *MiR-21* is the most studied microRNA in human cancer in which the upregulation has been commonly associated with the aggressive phenotype and unfavorable outcome (Pfeffer et al., 2015). However, limited studies have been performed to evaluate the expression levels of circulating *miR-21* and the biological correlation with treatment response and survival in breast cancer patietns. To develop novel non-invasive microRNA-based biomarkers in breast cancer, several approaches have been studied including those using plasma or serum (Sahlberg et al., 2015) and whole blood (Schrauder et al., 2012). As previously reported that plasma profile is able to detect any alteration after tumor surgery, plasma was used in this study. We investigated the potential role of circulating *miR-21* as a minimally-invasive marker for diagnostic and treatment monitoring marker in breast cancer. 

Jinling et al., (2017) and Wang et al., (2015) have performed meta-analysis of *miR-21* expression as prognostic marker involving more than 1,400 breast cancer patients and both studies have shown that upregulation of *miR-21* is correlated with shorter overall survival. Two studies (Müller et al., 2014; Wang et al., 2015) measured circulating *miR-21* expression in a total of 453 breast cancer patients and showed that the upregulation was significantly associated with shorter overall survival. In accordance with previous reports (Müller et al., 2014; Wang et al., 2015), our study showed that circulating *miR-21* expression was significantly higher in breast cancer patients compared to healthy women. We also primarily demonstrated that *miR-21* expression decreased significantly after surgery and chemotherapy rendering the potential role as therapeutic monitoring marker. Measuring in breast cancer patient’s whole blood, the previous study did not observe different *miR-21* expression measured before and after surgery (Alunni-Fabbroni et al., 2018). Alterations of microRNA expression after surgical resection were also not observed in the whole blood of patients with lung cancer (Patnaik et al., 2017). Different from plasma, whole blood contains both extracellular (within microvesicles, apoptotic bodies, and protein-bound) and intracellular (within blood cells) microRNAs that might influence to relevance to the presence of the tumor per se. As we found decreased levels of circulating *miR-21* after treatment, further study to related the magnitude of the reduction with clinical parameters and outcome. 

Expression levels of circulating *miR-21* were not significantly different among different clinical stages and tumor differentiation grades. Other previous studies (Alunni-Fabbroni et al., 2018; Gao et al., 2013) also did not find an association of *miR-21* expression with baseline stages and differentiation grades. We presented higher expression of circulating *miR-21* in young breast cancer patients (less than 40-year-old) that are commonly associated with aggressive biology and poor prognosis (Anwar et al., 2019). Although further study is required, our finding might reflect that *miR-21* upregulation is an important marker for diagnosis and therapeutic response. Several studies have shown the deregulation of *miR-21* expression in a subgroup of triple negative breast cancers (Bahrami et al., 2018; Shin et al., 2015) that have also been associated with more aggressive behavior and poor prognosis. Other studies have shown an association between *miR-21* upregulation (together with deregulation of other circulating microRNAs) with hormonal status (Wang et al., 2010), histopathology grades (Wang et al., 2010), and distant metastases (Asaga et al., 2011). We confirmed previous studies (Gao et al., 2013; Wang et al., 2015) by showing that upregulation of circulating *miR-21* at baseline is associated with lower progression-free survival in breast cancer. Although other studies (Chang et al., 2016) do not show the correlation with breast cancer prognosis, factors related to tumor heterogeneity, clinical presentation at diagnosis, and overall treatment have also influenced the disease outcome.

The major strength of this initial study is the additional evaluation of circulating *miR-21* expression after surgery and chemotherapy suggesting the value of a biomarker in response to the decreased tumor burden. However, upcoming study evaluating the magnitude of decreased *miR-21* expression and the association with various clinicopathological and clinical outcome of breast cancer patients is required. A constraint of this initial study is the variability of baseline clinical stages, treatment received, potential tumor heterogeneity, and relative small patient cohort. However, we showed that circulating *miR-21* expression at diagnosis was not significantly different according to various stages, tumor sizes, tumor differentiation, and breast cancer subtypes. Further study might address circulating *miR-21* expression in different breast cancer subtypes, response to specific chemotherapy regimens and hormonal therapy in a bigger / multicenter cohort. 

## Conflict of interest

All authors have declared for no financial and professional competing interests exist.
